# Engineering Epitaxial Silicene on Functional Substrates for Nanotechnology

**DOI:** 10.34133/2019/8494606

**Published:** 2019-09-12

**Authors:** Carlo Grazianetti, Alessandro Molle

**Affiliations:** CNR-IMM Unit of Agrate Brianza, Via C. Olivetti 2, Agrate Brianza I-20864, Italy

## Abstract

Two-dimensional materials are today a solid reality in condensed matter physics due to the disruptive discoveries about graphene. The class of the X-enes, namely, graphene-like single element artificial crystals, is quickly emerging driven by the high-momentum generated by silicene. Silicene, in addition to the graphene properties, shows up incidentally at the end of Moore's law debate in the electronic era. Indeed, silicene occurs as the crafted shrunk version of silicon long yearned by device manufacturers to improve the performances of their chips. Despite the periodic table kinship with graphene, silicene and the X-enes must deal with the twofold problem of their metastable nature, *i.e.*, the stabilization on a substrate and out of vacuum environment. Synthesis on different substrates and deep characterization through electronic and optical techniques of silicene in the early days have been now following by the tentative steps towards reliable integration of silicene into devices. Here, we review three paradigmatic cases of silicene grown by molecular beam epitaxy showing three different possible applications, aiming at extending the exploitation of silicene out of the nanoelectronics field and thus keeping silicon a key player in nanotechnology, just in a thinner fashion.

## 1. Introduction

**Figure 1:**
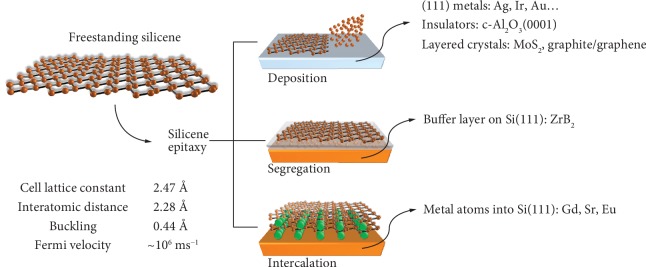
Scheme of the state-of-the-art for silicene production. Main properties of freestanding silicene are summarized on the left (data from reference [17]). Hitherto, silicene can be synthesized by deposition on substrates, segregation through a buffer layer, or by intercalation with metal atoms in bulk silicon (right). In the deposition framework, silicene is currently grown on (111)-terminated metals, insulators, and layered crystals.

Silicon is ubiquitously utilized in advanced technology at varying size scales. Mainly, it serves as a fundamental building block in mainstream digital electronics either as a platform for solid-state devices in a chip or as an active layer in flexible electronics. Nonetheless, despite the absence of an optical energy gap, it is also a key material for photovoltaics and photonics where silicon is used as a light absorber and an optical transport medium, respectively. Due to its versatility in applications, silicon is basically subjected to dimensional reduction addressing nanotechnology directions. Mastering a dimensional control of silicon is a key enabler for a wealth of urgent and exploratory functions. Not only silicon is currently forced down to the form of nanoscaled ultrathin body channels in high-performance field-effect transistors (FETs) [1], but silicon nanostructures such as a one-dimensional nanowire or nearly zero-dimensional nanocrystals have been widely investigated in terms of their inherent quantum properties and light-matter interaction [2]. All along this fashion, silicene is the two-dimensionally reduced form of silicon. Opposed to graphene, perfectly flat silicene is not permitted but stability is restored whenever a buckled atomic layout with mixed *sp*^2^ and *sp*^3^ bonds sets in. Within this canon, two-dimensional (2D) structures of silicon can be experimentally stabilized by forcing the silicon-silicon bonding to buckle while accommodating on a commensurate template or framework [3]. Inspired by pristine silicene models, the epitaxy of silicene on metal substrate has tracked the very first node of a silicene production roadmap that has been followed by other host configurations including 2D-layered crystals and insulating sapphire as illustrated in Figure 1. Early demonstration of silicene dates back to the epitaxy on (111)-terminated silver where silicon-silver surface commensurability along with the interplay of silicon and silver orbital was the key ingredients for silicene to set in [4]. While silver was used to host silicene multilayer [5], extension to other (111)-terminated metal substrates like iridium or gold [6, 7] was also reported for the molecular beam epitaxy (MBE) of a silicene single layer. The original approach was upgraded by the use of process-friendly silver-on-mica substrates allowing for the easy delamination of silicene single and multilayer and hence their integration into a transistor. Nearly on the same time, silicene was demonstrated as deriving from the segregation of silicon substrate atoms on top of a commensurate ZrB_2_ layer [8]. The first attempt to grow silicene on layered MoS_2_ compounds is in 2014 [9], more recently followed by the isolation of silicene domains on graphite [10]. These achievements offer a high potential to have silicene supported by easily handable flakes or nanosheets. Last but not the least, in 2018, reduction of a silicon film on a sapphire substrate down to the 2D edge was observed to result in Dirac-like electrodynamics that is suggestive of a silicene-sapphire coupling [11]. At the same time, an increasing effort is currently standing up on the synthesis of functionalized silicene where segregation from substrate is mediated by the atomically thin metal films (gadolinium, strontium, and europium) that are prone to host silicon in (layered) Zintl silicide structure [12] therein bearing a ferromagnetic behavior at the 2D level. This approach is somehow connected with silicene growth by intercalation in between a substrate and a secondary 2D layer enabling silicon atoms to pass through and position in between.

A common aspect for each reported configuration is the Al_2_O_3_ stabilizer [13]. This is a capping layer that immobilizes silicene in a sandwiched structure against any structural degradation or environmental reactivity when drawn outside the vacuum ambient. Currently, the choice of a support substrate is pivotal to determine either the viability of silicene handling processes in case of silicene transfer from its pristine site or the application target(s) whenever silicene is integrated into a device structure together with its substrate. In the former case, a delamination method was developed starting from epitaxial silicene-on-silver in order to make lone silicene operational as a transistor channel displaying an ambipolar transport behavior at room temperature (RT). Examples of the latter case are silicene-on-MoS_2_ and silicene-on-sapphire where the silicene-on-substrate structure is readily integrable into an application platform with no transfer processing due. Our purpose here is to outline significant directions for silicene epitaxy in order to envisage future nanotechnology exploitation. These ones will be compared with other emerging approaches to the epitaxy of silicene or functionalized silicene. Finally, open challenges for viable silicene nanotechnologies will be stated.

### 1.1. Universal Silicene Encapsulation/Stabilization

**Figure 2:**
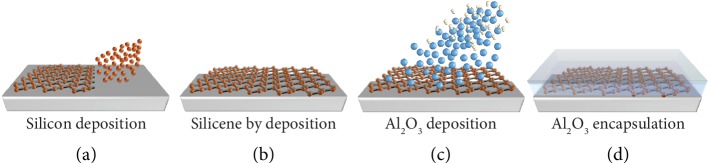
Schematics of the sequential steps for the Al_2_O_3_ encapsulation of epitaxial silicene. Silicon deposition by MBE (a); stabilization via interaction with substrate of metastable silicene (b); reactive molecular beam deposition of Al_2_O_3_ by aluminum flux in O_2_-rich overpressure (c); “silicene sandwich” where silicene is stabilized either on top or at the bottom (d).

While accommodating on substrates, silicene quickly degrades from its top face if exposed to environmental conditions. Degradation results in the silicon oxidation arising from direct incorporation of oxygen atoms in between the silicon bonds or amorphization-mediated oxygen reactions [13]. Both cases are related to the higher reactivity of the remnant *sp*^3^ hybrid bonds that are constitutional of the buckled silicene structure. Sequential encapsulation with a nonreactive capping layer of amorphous Al_2_O_3_ is an effective way to prevent silicon from degradation and preserve its own structure. Al_2_O_3_ films are grown from RT reactive deposition of aluminum atomic flux in O_2_-rich overpressure. Predeposition of an aluminum ultrathin layer prevents silicon from getting locally oxidized and serves as precursor for the successive Al_2_O_3_ layers to grow up. Protection and stabilization of the silicene layer are validated by *in situ* X-ray photoemission spectroscopy of the Si 2*p* core level (before and after encapsulation) and *ex situ* Raman spectroscopy of the encapsulated structure. The former one makes sure that the chemical nature of the silicene bonding is not affected by Al_2_O_3_ capping [13], while the latter one appears as a quick probe of the characteristic Raman spectrum of silicene [14]. This encapsulation strategy is pictorially illustrated in Figure 2 for the case of epitaxial silicene grown on a delaminable substrate, for instance epi-Ag(111)-on-mica. It can be applied to each silicene configuration, therein proving a general environmental stabilization strategy whenever the encapsulated layer is taken out from the vacuum. Moreover, the effectiveness of the same can be extended potentially to other silicene-like X-enes like stanene or epitaxial phosphorene [15, 16].

## 2. Discussion

### 2.1. Silicene-on-Silver

**Figure 3:**
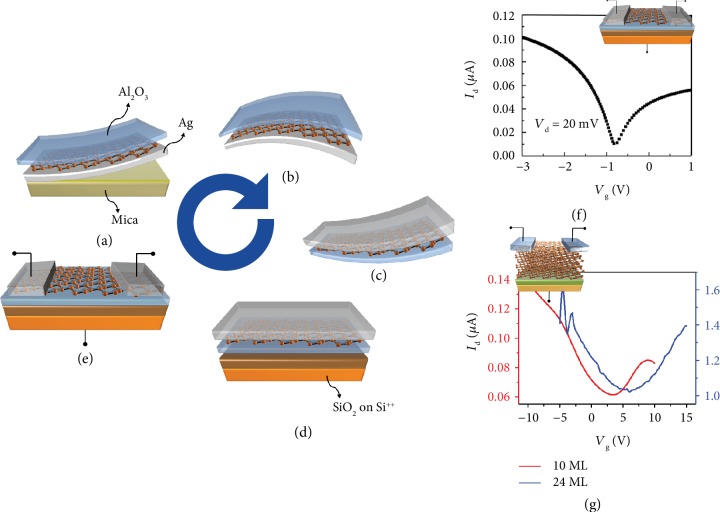
Silicene on Ag(111). Silicene grown on thin film silver on mica substrate and then encapsulated with Al_2_O_3_ stabilizer (a); mica removal resulting in a thin (~300 nm) membrane (b); the “silicene sandwich” is flipped upside down (c) and placed onto a new supporting substrate like SiO_2_/Si^++^ (d); finally, source and drain electrodes are patterned on the native silver layer and residual silver is etched away (e). Electrical characterization of the silicene-based FETs made of monolayer (f) and multilayer (g) channels showing an ambipolar behavior at RT. (f) and (g) are adapted from references [19, 21].

Silicene on Ag(111) represented a decisive milestone in the development of the X-enes beyond graphene [4]. It was the first time that silicon was demonstrated to arrange in a nonconventional crystal lattice when shrunk down in 2D, with silicon bonds showing a mixed hybridization in between the *sp*^2^ of flat graphene and the *sp*^3^ of diamond. Many experimental studies corroborated by theoretical support constitute now the advanced knowledge on this artificial 2D crystal. Hitherto, Ag(111) is probably the most widely used template for silicene. Therefore for an extended discussion of the physical and chemical properties of silicene on Ag(111), we refer to detailed topic reviews [3, 17]. Here, we will focus on the specific route implemented to bypass the role of Ag(111) substrate for integrating silicene into electronic devices despite its pristine growth on a metal substrate. This route is therefore specific for silicene grown by MBE on metal substrates, even if it might be also adapted to silicene achieved by intercalation or segregation methods (see Figure 1) with appropriate measures [3]. On Ag(111), silicene cannot be grown in its freestanding fashion, *i.e.*, with silicon atoms alternatively up and down, but lies down by accommodating its silicon atoms on top or in between the silver ones. The resulting silicene sheets, *i.e.*, 3 × 3, √7×√7, and √7×√7 freestanding silicene reconstructions (or equivalently 4 × 4, √13×√13, and 2√3 × 2√3 silver superstructures), constitute a phase diagram on monocrystalline silver substrate that can be equally resumed on thin silver epitaxial films displaying (111) termination [18]. The silver substrate plays the same key role in dictating the details of the multilayer silicene growth, namely, the deposition of a multiple silicene layer on top of the pristine one through a terrace growth mode [5]. Indeed, only when the single layer silicene acts as a seeding layer for the additional silicene layers in a three-dimensional (3D) growth regime, the multilayer silicene shows up with both clear √3×√3 termination and a characteristic Raman shift markedly different from those of conventional crystalline and amorphous silicon [19]. Interestingly, the oxygen intercalation through bilayer silicene is a viable route to achieve quasi-freestanding silicene decoupled from the silver substrate which survives in the ambient environment for at least 120 hours [20]. The thickness reduction of the silver template down to thin films, either in the form of epitaxial silver on mica [21] or on Si(111) [18] substrates, appeared as the enabling step for the exploitation of silicene in applicative directions like the integration into a FET device as detailed in the following. For instance, thin epitaxial silver on mica bypasses the use of highly expensive monocrystal substrates and allows for the delamination of fully encapsulated silicene heterostack out of the solid mica substrate. Even reduced to the nanometer thickness, silver still presents two technical hurdles. The former is related to the removal of the rigid template, *e.g*., silicon or mica, and the latter is concerned with the unavoidable contribution to the electronic transport of a thin silver film overwhelming silicene, when integrated as a FET channel. Mica can be mechanically delaminated from the silver thin film by means of a cutter-assisted two-tape method allowing for a large area (few cm^2^) transfer of the thin silver film (Figure 3(a)). All along this way, a thin membrane where silicene is sandwiched in between the residual silver at the bottom and the Al_2_O_3_ stabilizer (Figure 3(b)). Then, the so-achieved flexible membrane can be faced downward onto a new supporting substrate, *e.g.*, a device substrate like SiO_2_/Si^++^ (Figures 3(c) and 3(d)). This flip exposes the Al_2_O_3_ in contact with device substrate and the silver layer to air. A key point at this stage is to recycle the native silver layer in source and drain contacts by taking benefit from the known hybridization between it and the originally supported silicene (see Figure 3(e)). By means of electron beam lithography, the silver native layer can be patterned in a FET configuration by designing silver-made contacts and silver-free active channel. In addition to silver-made source and drain contacts, the shrewdness of using silicon oxide on highly doped silicon readily serves as bottom gate. Remarkably, the same process flow equivalently stands out both for single and for multilayer silicene. Single and multilayer silicene-based FETs in low-field (*V*_d_ = 20 mV) conditions show an ambipolar transport behavior at RT (Figures 3(f) and 3(g)) [19, 21]. In order to numerically characterize these FETs, the well-accepted ambipolar model used for graphene FET [22] has been used as well. Fitting the resistance (*R* = *V*_d_/*I*_d_) plots, the best mobility values achieved for mono- and multilayer silicene FETs are 100 and 200 cm^2^/Vs, respectively. Although below the maximum value predicted by the theory for freestanding/ideal silicene, *i.e.*, 1000 cm^2^/Vs [23], these mobility values are however encouraging in perspective as they are prone to further optimization. On the one hand, silicene FETs suffer from stability issues that have to be fixed by means of a second stage encapsulation on the silver-free silicene channel (see Figure 3(e)), possibly leading to enhanced mobility through dielectric screening effects [24]. On the other hand, silicene itself can be perfectioned in its inherent structure by reducing the density of grain boundaries or point defects (namely, unavoidable by-products of the epitaxy) that act as mobility limiter. Despite these further and necessary improvements, silicene FETs turn out to be a disruptive proof-of-concept paving the way to viable process schemes towards reliable silicene integration.

### 2.2. Silicene-on-MoS_2_

**Figure 4:**
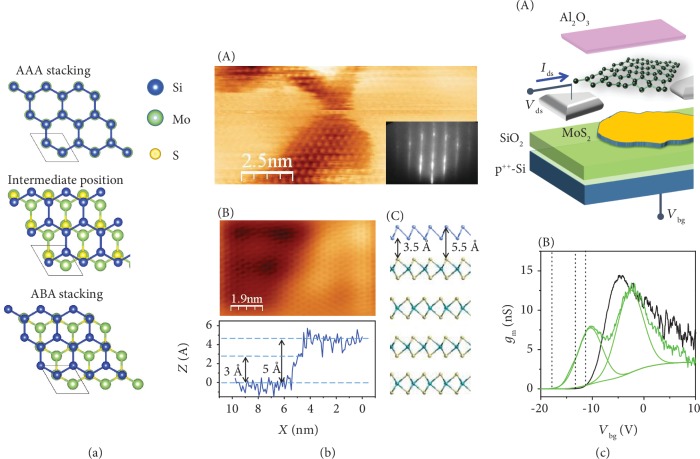
Silicene on MoS_2_. Stacking models for silicene on MoS_2_ (a); STM topography of silicene on MoS_2_ (inset: reflection high-energy electron diffraction figure) (A), atomically resolved topography of silicene on MoS_2_ at the step-edge (B) and related height profile, a cross-sectional model of AAA-stacked silicene-on-MoS_2_ (C) (b); pictorial sketch of the heterointerface-based FET composed by Al_2_O_3_/silicene/MoS_2_ heterosheet on SiO_2_/Si^++^ substrate (A) and its transconductance plot (B) (c). Panels are adapted from references [9, 29].

Structures composed by alternating semimetallic and semiconducting (or insulating) 2D materials are very interesting for use in nanoelectronic devices as they may work as a prototypical device structure triggering different electronic and optoelectronic characteristics [25]. These so-called “van der Waals (vdW) heterostructures” are composed by 2D layers in which the strong covalent bonds provide the in-plane stability of the 2D layers, whereas relatively weak (vdW) forces are sufficient to keep the layers together [26]. Inspired by this material concept, here, we outline the case of the silicene epitaxy on MoS_2_ surface as derived from rheological pieces and mechanically exfoliated flakes. MoS_2_ has become extremely popular as the first representative of layered transition metal dichalcogenides with a semiconducting character. Computational outputs based on density functional theory select three different silicene configurations that are commensurate with the MoS_2_ surface lattice in terms of silicon atom accommodation as sketched in Figure 4(a) [9]. These include one-by-one atom match (AAA stacking), alternate atom match (ABA stacking), and an intermediate positioning of the silicene lattice. Match with the experimental facts results in a highly buckled silicene lattice with AAA stacking. Basically, this picture comes from the positional correspondence of silicon atoms in the silicene lattice with protruding sulphur atoms of the MoS_2_ top layer as deduced from scanning tunneling microscopy (STM) imaging (see Figure 4(b)). In Figure 4(b), the top one can clearly discriminate MoS_2_ regions from silicene ones while ruling out any amorphous silicon formation as from the electron diffraction figure in the inset. A more detailed insight into atomically resolved topography (Figure 4(b), B) reveals a one-by-one positional correspondence between top Si atoms and substrate surface atoms and two characteristic heights at the MoS_2_-silicene step-edge, 3 and 5 Å. Both facts are consistent with highly stretched silicon bonding with a AAA stacking (Figure 4(b), C). In agreement with the computational modelling of the electronic bands, so-grown silicene proves to be metallic in character as resulting from direct evidence of the local density of states [9]. Recently, this picture has been objected by figuring out silicon intercalation in between the upmost adjacent MoS_2_ layers [27]. Intercalation of silicon atoms inside layered materials to form a self-organized silicene layer is an emerging topic of study in a number of combinations; however, many factors in the silicon growth processing as well as in the affinity of silicon with underlying substrate may affect the details or the emergence of the intercalation. This scenario is well-described by the case of silicene intercalated in between graphene and ruthenium by silicon deposition on top where the growth temperature triggers silicon atoms from being arranged as bubble to a silicene lattice below the graphene layer [28]. When grown on freshly recovered MoS_2_ flakes at 200°C, the highly buckled silicene lattice proves super-surface positioning based on compositional depth sensitive diagnostic. Subsequently, it was capped by Al_2_O_3_ stabilizer and integrated into a FET structure patterned on a Al_2_O_3_/silicene/MoS_2_ heterosheet. This heterosheet was in turn supported by a SiO_2_/Si^++^ substrate serving as a bottom gate electrode as well as for the above-mentioned silicene FET [29]. Despite (high-buckled) silicene being metallic, the transfer characteristics display a field-modulated double transconductance peak as shown in Figure 4(c). The twofold feature was ascribed to two separate transport channels, one driven by the field drop at the MoS_2_/SiO_2_ interface and another at the silicene/MoS_2_ interface [29]. The feasibility of such silicene-based heterosheet transistor paves the way to other device analogues where appropriate layered materials can be used to bear silicene without substrate decoupling therein addressing a stability issue in the process flow.

### 2.3. Silicene-on-Sapphire

**Figure 5:**
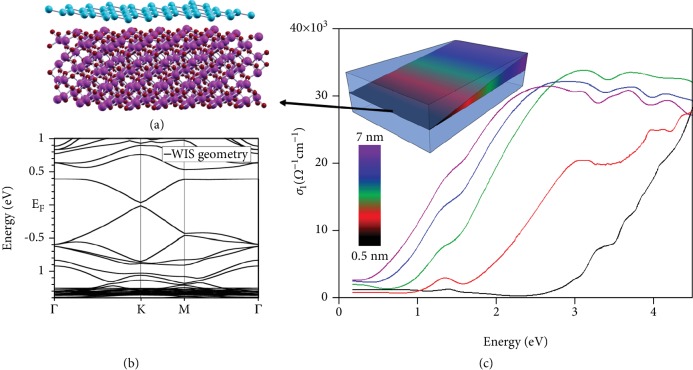
Silicene on sapphire. A theoretical model of the weakly interacting silicene on Al_2_O_3_(0001) substrate (a); its electronic band structure (b); and the optical conductivity of the silicon nanosheets of variable thickness from 0.5, *i.e.*, silicene, to 7 nm as schematically depicted in the inset (c). Panels are adapted from reference [11].

The proved dramatic interaction between the metal substrate and silicene drove the theoretical efforts towards a refined survey of template compatible with silicene [30]. In this sense, not only lattice-match conditions (verified by relaxing the honeycomb structure) are taken into account but also the band alignment between the silicon overlayer and the substrate. Silicene (also germanene) can be proved to be stabilized by Al_2_O_3_(0001) as substrate in a reconstructed fashion with respect to the freestanding lattice [30]. First principle calculations prove that silicene is stable when formed on the Al-terminated surface exhibiting a low-buckled honeycomb structure with gapped Dirac cones at K point. However, even on Al_2_O_3_(0001), the role of interaction turns out to tune the electronic band structure [11]. Indeed, among the manifold minima of the Born-Oppenheimer energy, it is possible to identify two groups of silicene geometries that differentiate each other about the degree of interaction with the Al_2_O_3_(0001) surface. Although their common structure is a metastable √13×√13 silicene lattice on the 3 × 3 Al_2_O_3_(0001) substrate (Figure 5(a)), accounting for a strain of about 3.3% because of the lattice mismatch, when the average distance between the silicene and substrate is about 2.8 Å (3.3 Å), the interaction is strong (weak). In both cases, being a reconstruction of the freestanding lattice, either weak (W) or strong interacting silicene (SIS) lose its pristine D_3d_ symmetry. Although WIS and SIS configurations are separated by a small energy difference, their electronic and optical properties are remarkably different. Figure 5(b) shows the electronic band structure of the WIS geometry showing small bandgap opening in the Dirac cone of about 0.05 eV at K point retaining the linear band behavior of freestanding silicene close to the Fermi level. Conversely, the SIS geometry is characterized by a larger (indirect) bandgap opening without features of the Dirac energy dispersion (not shown). At variance with metal substrate, *e.g.*, Ag(111) [31], the Al_2_O_3_(0001) substrate intriguingly paves the way for the first time optical characterization of silicene [11]. Opposed to relatively cold growth of silicene-on-silver, silicene is grown on a Al_2_O_3_(0001) surface at 670°C. Generally, silicon nanosheets grown on Al_2_O_3_(0001) exhibit a peculiar optical behavior as demonstrated in Figure 5(c). Similar to previously described cases, air-instability plagues the survival of silicene and thicker silicon film on Al_2_O_3_(0001); therefore, encapsulation with the Al_2_O_3_ is still necessary as described for the two previous cases in point while unaffecting the optical response of silicon due to its transparency in a large range of the electromagnetic spectrum. Hence, the silicon nanosheets are sandwiched in between two optical transparent layers for months without chemical intermixing [11]. By making tailored samples with variable silicon thickness along one substrate direction (see sketch in Figure 5) with a wedge-like shape, the overall silicon nanosheet thickness scrutinized in our survey ranges between 0.5 and 7 nm. The real part of the optical conductivity *σ*_1_, extracted from transmittance measurements through Kramers-Kronig transformations, is shown in Figure 5(c). In the photon range 0.25-4.5 eV, the optical conductivity *σ*_1_ shows a thickness-dependent behavior. At the lowest thickness investigated at the pure 2D limit, *i.e.*, 0.5 nm, the black curve of Figure 5(c) is characterized by two spectral features at ~1.4 and 4.5 eV. These peaks closely resemble those arising from the resonant interband transitions *π* → *π*^∗^ and *σ* → *σ*^∗^ occurring at the M and Γ points of the Brilluoin zone of freestanding silicene due to the van Hove singularities of the joint density of states [32]. These spectral features are the characteristic hallmarks of the X-enes distinguishing graphene, silicene, and germanene, while they share the low-energy part of the spectrum with the same absorption behavior depending only on *πα*, where *α* is the Sommerfeld fine-structure constant, linking the quantum electrodynamics to the condensed matter physics [33]. Noticeably, these interband transitions are demonstrated to occur in the WIS geometry. In particular, the pristine *π* → *π*^∗^ transition of silicene is almost unaffected by the presence of Al_2_O_3_(0001) substrate, at variance with Ag(111) [31], and by the strain, thus being very close to the experimental one. When the silicon nanosheet thickness is increased up to the maximum thickness of 7 nm, the optical behavior progressively deviates from that of freestanding silicene but still remains unconventional and does not recover the behavior of bulk amorphous or crystalline silicon because of the absence of an optical gap. On the other hand, the silicene-like nature of the thinnest silicon nanosheet investigated is further corroborated by an additional evidence. The low-energy part of the *σ*_1_ reported in Figure 5(c) can be demonstrated to be quantized as a multiple integer of *π*e^2^/2*h*, *i.e.*, the universal optical conductance G_0_ [34], where *h* is the Planck constant. In other words, the two silicon nanosheets with thickness of 0.5 and 1.5 nm show a low-energy optical conductance equal to G_0_ and 2G_0_, respectively. This is an additional evidence of the presence of massless Dirac electrons as already reported on single and few-layer graphene [35]. With the exception of the inelastic scattering associated with phonons, *i.e.*, Raman spectroscopy, metal substrates hamper to probe the optical properties of silicene. Therefore, the choice of the transparent Al_2_O_3_(0001) for the theoretically predicted weakly interacting silicene allowed for the first time measured optical properties by fabricating and encapsulating silicon nanosheets with variable thickness on Al_2_O_3_(0001). A Dirac-like absorption is observed in the infrared range of the optical conductivity at the 2D limit. This optical conductivity shows an overall behavior similar to that expected from the ideal silicene with a clear feature related to the *π* → *π*^∗^ interband transition that is corroborated by the evidence of a quantized optical conductance for the first two layers. This outcome opens up the opportunity to exploit graphene-like silicon in a silicon photonics directions operating in the THz regime.

## 3. Conclusion

The epitaxy on substrate is the methodological pathway to make silicene a real material beyond the concept, provided that the substrate is carefully selected under stringent requirements of commensurability and some degree of orbital hybridization. Although the synthesis of freestanding silicene would represent a decisive breakthrough for fundamental research, on the other hand, the fabrication of silicene on supporting substrates is a decisive technological outcome in the nanotechnology roadmap nodes. In this framework, the three options outlined here, namely, silicene-on-silver, silicene-on-MoS_2_, and silicene-on-sapphire, define cases in point where silicene can be readily implemented for applications. The first two cases target nanoelectronics, one pointing to a purely silicene-based FET, the other to a general heterointerface device coupling different 2D materials. The third case is the first example of a silicene-on-insulator canon which paves the way to silicene application to photonics, a field that has been completely unexplored so far, but that can strikingly benefit from responsivity of an ultrascaled silicon block in the THz or infrared spectrum. Nonetheless, several bottlenecks still remain as to what kind of stabilization and integration processing should be developed to have a durable function from silicene as active part in a device. Clearly, silver-free silicene is prone to fast degradation in environment. This drawback demands for additional encapsulation after detaching silicene from its native silver substrate or alternative passivation approaches enabling to disentangle silicene from its substrate. In this framework, a key role will be played by the development of 2D insulators beyond hexagonal BN, *e.g.*, serving as ultrathin gate insulators for devices [36]. Once a full stabilization in a device is defined, transport features in silicene are still an open field to investigate fundamental properties, such as quantum phenomena, as well as technology improvements. On the other way around, when silicene is grown on MoS_2_ and sapphire, substrate detachment is not necessary and new scenarios for applications show up. On the former case, silicene would constitute a building block to expand the portfolio of vdW heterostructures with the benefit of adapting its structure to support 2D layer. On the latter case, stable silicene confined in between two optically transparent and protective layers can be readily shifted to a plasmonic grating to check a resonance effect in the THz regime as reported for graphene [37]. In all the discussed cases, the future standardization of reliable integration processes will definitely assess the role of the X-enes in nanotechnology. The ongoing efforts on silicene and the X-enes should not be therefore focused only on silicene itself but also and mostly on the functional coupling with substrate to be an active part, and not merely support, for applications in nanoelectronics, flexible electronics, topological physics, optoelectronics, and photonics.
